# 4-[(*E*)-({4-[(4-Amino­phen­yl)sulfon­yl]phen­yl}imino)­meth­yl]phenol ethanol monosolvate

**DOI:** 10.1107/S1600536812021563

**Published:** 2012-05-19

**Authors:** Sadaf Afzal, Zareen Akhter, M. Nawaz Tahir

**Affiliations:** a Department of Chemistry, Quaid-i-Azam University, Islamabad, Pakistan; b University of Sargodha, Department of Physics, Sargodha, Pakistan

## Abstract

In the title compound, C_19_H_16_N_2_O_3_S·C_2_H_6_O, the 4-hy­droxy­benzyl­idene group is oriented at dihedral angles of 73.17 (7) and 77.06 (7)° with respect to the aniline groups. The sulfonyl group make dihedral angles of 44.89 (13) and 59.16 (12)° with the adjacent aniline groups. In the crystal, a two-dimensional polymeric network parallel to (010) is formed by N—H⋯O, O—H⋯N and O—H⋯O hydrogen bonds. There also exist π–π inter­actions with a distance of 3.5976 (18) Å between the centroids of hy­droxy­phenyl rings.

## Related literature

For related structures, see: Bocelli & Cantoni (1990 ▶).
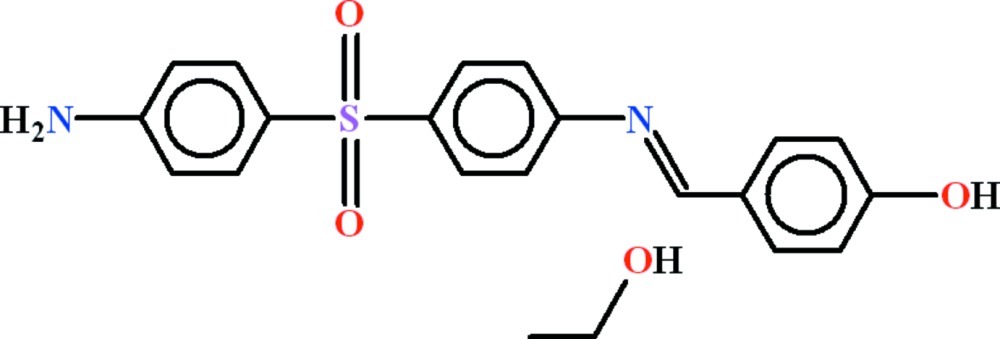


## Experimental

### 

#### Crystal data

C_19_H_16_N_2_O_3_S·C_2_H_6_O
*M* *_r_* = 398.47
Monoclinic, 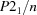

*a* = 8.5281 (3) Å
*b* = 25.3057 (12) Å
*c* = 9.4084 (4) Å
β = 96.738 (3)°
*V* = 2016.40 (15) Å^3^
*Z* = 4
Mo *K*α radiation
μ = 0.19 mm^−1^
*T* = 296 K
0.35 × 0.25 × 0.20 mm

#### Data collection

Bruker Kappa APEXII CCD diffractometer
Absorption correction: multi-scan (*SADABS*; Bruker, 2005 ▶) *T*_min_ = 0.948, *T*_max_ = 0.968
16497 measured reflections
3969 independent reflections
2400 reflections with *I* > 2σ(*I*)
*R*_int_ = 0.039

#### Refinement

*R*[*F* ^2^ > 2σ(*F* ^2^)] = 0.054
*wR*(*F* ^2^) = 0.138
*S* = 1.04
3969 reflections
256 parameters
H-atom parameters constrained
Δρ_max_ = 0.22 e Å^−3^
Δρ_min_ = −0.22 e Å^−3^

### 

Data collection: *APEX2* (Bruker, 2009 ▶); cell refinement: *SAINT* (Bruker, 2009 ▶); data reduction: *SAINT*; program(s) used to solve structure: *SHELXS97* (Sheldrick, 2008 ▶); program(s) used to refine structure: *SHELXL97* (Sheldrick, 2008 ▶); molecular graphics: *ORTEP-3 for Windows* (Farrugia, 1997 ▶) and *PLATON* (Spek, 2009 ▶); software used to prepare material for publication: *WinGX* (Farrugia, 1999 ▶) and *PLATON*.

## Supplementary Material

Crystal structure: contains datablock(s) global, I. DOI: 10.1107/S1600536812021563/bq2358sup1.cif

Structure factors: contains datablock(s) I. DOI: 10.1107/S1600536812021563/bq2358Isup2.hkl

Supplementary material file. DOI: 10.1107/S1600536812021563/bq2358Isup3.cml

Additional supplementary materials:  crystallographic information; 3D view; checkCIF report

## Figures and Tables

**Table 1:** Hydrogen-bond geometry (Å, °)

*D*—H⋯*A*	*D*—H	H⋯*A*	*D*⋯*A*	*D*—H⋯*A*
O1—H1⋯O4^i^	0.82	1.89	2.698 (3)	171
N2—H2*A*⋯O3^ii^	0.86	2.30	3.105 (3)	156
N2—H2*B*⋯O2^iii^	0.86	2.21	3.026 (3)	157
O4—H4⋯N1^iv^	0.82	2.13	2.926 (3)	162

Symmetry codes: (i) 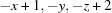; (ii) 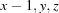; (iii) 
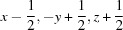
; (iv) 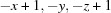.

**Fig. 1.**
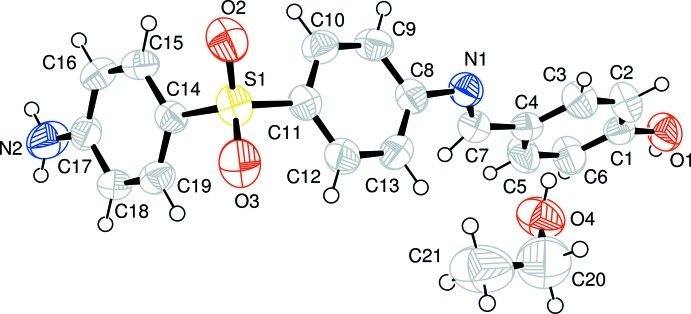
View of the title compound with displacement ellipsoids are drawn at the 50% probability level.

**Fig. 2.**
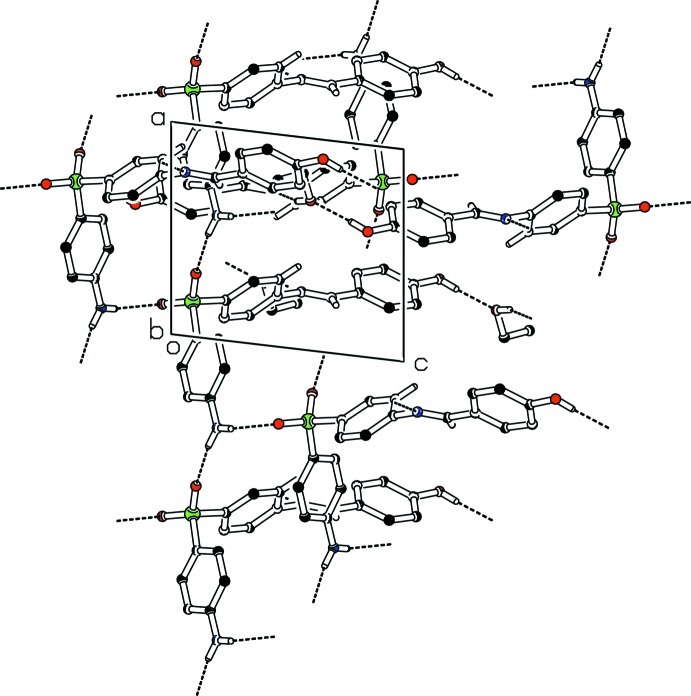
The partial packing (PLATON; Spek, 2009) which shows that molecules form two dimensional polymeric network. The H-atoms not involved in H-bondings are omitted for clarity.

## supplementary crystallographic information

### Comment

The structure of 4,4'-diamino-diphenylsulfone (Bocelli & Cantoni, 1990), related to the title compound, (I), shown in Fig. 1., has been published previously. In (I), the 4-hydroxybenzaldehyde moiety A (O1/C1–C7), the anilinic moieties of 4,4'-diaminodiphenylsulfone B (N1/C8—C13) and C (C14—C19/N2) are planar with r.m.s. deviation of 0.0125 Å, 0.0320 Å and 0.0151 Å, respectively. The dihedral angles between A/B, A/C and B/C are 73.17 (7)°, 77.06 (7)° and 77.16 (7)°, respectively. The sulfonyl group D (O2/S1/O3) is of course planar. The dihedral angles between B/D and C/D are 59.16 (12)° and 44.89 (13)°, respectively. The molecules are stabilized in the form of two-dimensional polymeric network due to various type of H-bondings (Table 1, Fig. 2). There exist also π–π interaction between the CgA···CgA^i^ [i = 1 - *x*,-*y*, 2 - *z*] at a distance of 3.5976 (18) Å, where CgA is the centroid of phenyl ring (C1—C6).

### Experimental

In a 250 ml two-necked round bottomed flask equipped with condenser and magnetic stirrer, 4-hydroxy benzaldehyde (1.22 g, 0.01 mole) was dissolved in 50 ml of dried ethanol under inert atmosphere of nitrogen gas. 4,4'-diaminodiphenylsulfone (1.24 g, 0.005 mole) was added to it. The reaction mixture was refluxed for 6 h with constant stirring, and the progress of the reaction was monitored by TLC [n-hexane/ethanol (3:1)] respectively. The yellow colored product thus obtained was filtered, dried and hence recrystallized in ethanol. Yield: 88%, m.p.: 385 K.

### Refinement

The H-atoms were positioned geometrically (C—H = 0.93–0.97 Å, N—H = 0.86 Å O—H = 0.82 Å) and refined as riding with *U*_iso_(H) = *xU*_eq_(C, N, O), where *x* = 1.5 for methyl groups and *x* = 1.2 for other H atoms.

### Crystal data

**Table:**

C_19_H_16_N_2_O_3_S·C_2_H_6_O	*F*(000) = 840
*M**_r_* = 398.47	*D*_x_ = 1.313 Mg m^−^^3^
Monoclinic, *P*2_1_/*n*	Mo *K*α radiation, λ = 0.71073 Å
Hall symbol: -P 2yn	Cell parameters from 2522 reflections
*a* = 8.5281 (3) Å	θ = 1.6–26.0°
*b* = 25.3057 (12) Å	µ = 0.19 mm^−^^1^
*c* = 9.4084 (4) Å	*T* = 296 K
β = 96.738 (3)°	Prism, yellow
*V* = 2016.40 (15) Å^3^	0.35 × 0.25 × 0.20 mm
*Z* = 4	

### Data collection

**Table:**

Bruker Kappa APEXII CCD diffractometer	3969 independent reflections
Radiation source: fine-focus sealed tube	2400 reflections with *I* > 2σ(*I*)
Graphite monochromator	*R*_int_ = 0.039
Detector resolution: 8.00 pixels mm^-1^	θ_max_ = 26.0°, θ_min_ = 1.6°
ω scans	*h* = −10→10
Absorption correction: multi-scan (*SADABS*; Bruker, 2005)	*k* = −28→31
*T*_min_ = 0.948, *T*_max_ = 0.968	*l* = −11→11
16497 measured reflections	

### Refinement

**Table:**

Refinement on *F*^2^	Primary atom site location: structure-invariant direct methods
Least-squares matrix: full	Secondary atom site location: difference Fourier map
*R*[*F*^2^ > 2σ(*F*^2^)] = 0.054	Hydrogen site location: inferred from neighbouring sites
*wR*(*F*^2^) = 0.138	H-atom parameters constrained
*S* = 1.04	*w* = 1/[σ^2^(*F*_o_^2^) + (0.0486*P*)^2^ + 0.7969*P*] where *P* = (*F*_o_^2^ + 2*F*_c_^2^)/3
3969 reflections	(Δ/σ)_max_ < 0.001
256 parameters	Δρ_max_ = 0.22 e Å^−^^3^
0 restraints	Δρ_min_ = −0.22 e Å^−^^3^

### Special details

**Table:**

Geometry. Bond distances, angles *etc*. have been calculated using the rounded fractional coordinates. All su's are estimated from the variances of the (full) variance-covariance matrix. The cell e.s.d.'s are taken into account in the estimation of distances, angles and torsion angles
Refinement. Refinement of *F*^2^ against ALL reflections. The weighted *R*-factor *wR* and goodness of fit *S* are based on *F*^2^, conventional *R*-factors *R* are based on *F*, with *F* set to zero for negative *F*^2^. The threshold expression of *F*^2^ > σ(*F*^2^) is used only for calculating *R*-factors(gt) *etc*. and is not relevant to the choice of reflections for refinement. *R*-factors based on *F*^2^ are statistically about twice as large as those based on *F*, and *R*- factors based on ALL data will be even larger.

### Fractional atomic coordinates and isotropic or equivalent isotropic displacement parameters (Å^2^)

**Table:**

	*x*	*y*	*z*	*U*_iso_*/*U*_eq_	
S1	0.16578 (8)	0.17666 (3)	0.09119 (8)	0.0587 (3)	
O1	0.4083 (3)	−0.10515 (8)	1.1540 (2)	0.0809 (9)	
O2	0.1370 (3)	0.14540 (8)	−0.0375 (2)	0.0721 (8)	
O3	0.2983 (2)	0.21201 (8)	0.1066 (2)	0.0758 (8)	
N1	0.2707 (3)	0.01949 (9)	0.5599 (3)	0.0605 (9)	
N2	−0.4161 (3)	0.28543 (11)	0.1989 (3)	0.0851 (11)	
C1	0.3681 (3)	−0.07205 (11)	1.0424 (3)	0.0588 (11)	
C2	0.4236 (4)	−0.08302 (12)	0.9138 (4)	0.0721 (14)	
C3	0.3891 (4)	−0.05033 (12)	0.7990 (3)	0.0682 (12)	
C4	0.2988 (3)	−0.00543 (11)	0.8088 (3)	0.0562 (10)	
C5	0.2454 (4)	0.00539 (13)	0.9378 (3)	0.0764 (14)	
C6	0.2771 (4)	−0.02806 (13)	1.0536 (3)	0.0763 (14)	
C7	0.2612 (3)	0.03107 (11)	0.6889 (3)	0.0609 (11)	
C8	0.2394 (3)	0.05957 (11)	0.4557 (3)	0.0555 (10)	
C9	0.1327 (3)	0.04922 (12)	0.3366 (3)	0.0638 (11)	
C10	0.1075 (3)	0.08533 (11)	0.2278 (3)	0.0602 (11)	
C11	0.1906 (3)	0.13213 (10)	0.2355 (3)	0.0514 (10)	
C12	0.2935 (3)	0.14365 (12)	0.3551 (3)	0.0653 (11)	
C13	0.3182 (3)	0.10741 (12)	0.4654 (3)	0.0659 (11)	
C14	−0.0044 (3)	0.21204 (10)	0.1125 (3)	0.0484 (9)	
C15	−0.1499 (3)	0.19453 (11)	0.0475 (3)	0.0565 (10)	
C16	−0.2849 (3)	0.21927 (12)	0.0754 (3)	0.0595 (11)	
C17	−0.2800 (3)	0.26199 (11)	0.1685 (3)	0.0548 (10)	
C18	−0.1330 (3)	0.28033 (11)	0.2303 (3)	0.0556 (10)	
C19	0.0020 (3)	0.25527 (11)	0.2027 (3)	0.0561 (10)	
O4	0.7066 (3)	0.07876 (9)	0.6003 (2)	0.0849 (10)	
C20	0.8298 (5)	0.11197 (18)	0.5671 (5)	0.1181 (19)	
C21	0.7841 (6)	0.15143 (18)	0.4597 (5)	0.134 (2)	
H1	0.37154	−0.09407	1.22496	0.0971*	
H2	0.48515	−0.11294	0.90520	0.0862*	
H2A	−0.50591	0.27377	0.16033	0.1019*	
H2B	−0.41223	0.31187	0.25664	0.1019*	
H3	0.42698	−0.05840	0.71280	0.0817*	
H5	0.18667	0.03587	0.94737	0.0913*	
H6	0.23672	−0.02068	1.13905	0.0918*	
H7	0.22811	0.06497	0.70918	0.0731*	
H9	0.07762	0.01743	0.33023	0.0765*	
H10	0.03426	0.07826	0.14877	0.0723*	
H12	0.34661	0.17582	0.36174	0.0784*	
H13	0.38791	0.11523	0.54620	0.0789*	
H15	−0.15513	0.16594	−0.01499	0.0678*	
H16	−0.38177	0.20736	0.03146	0.0714*	
H18	−0.12709	0.30965	0.29026	0.0667*	
H19	0.09933	0.26744	0.24512	0.0673*	
H4	0.69645	0.05409	0.54350	0.1018*	
H20A	0.91206	0.09020	0.53444	0.1417*	
H20B	0.87447	0.12966	0.65393	0.1417*	
H21A	0.74450	0.13442	0.37156	0.2008*	
H21B	0.87416	0.17265	0.44508	0.2008*	
H21C	0.70331	0.17353	0.49098	0.2008*	

### Atomic displacement parameters (Å^2^)

**Table:**

	*U*^11^	*U*^22^	*U*^33^	*U*^12^	*U*^13^	*U*^23^
S1	0.0582 (4)	0.0594 (5)	0.0613 (5)	0.0032 (4)	0.0187 (3)	0.0088 (4)
O1	0.1062 (18)	0.0712 (14)	0.0637 (15)	0.0133 (12)	0.0030 (13)	0.0100 (12)
O2	0.0955 (15)	0.0701 (13)	0.0547 (14)	0.0147 (11)	0.0260 (11)	0.0005 (11)
O3	0.0545 (12)	0.0784 (14)	0.0973 (17)	−0.0049 (10)	0.0206 (11)	0.0223 (12)
N1	0.0746 (16)	0.0550 (15)	0.0515 (17)	0.0026 (12)	0.0056 (12)	0.0024 (13)
N2	0.0591 (16)	0.099 (2)	0.094 (2)	0.0173 (14)	−0.0038 (14)	−0.0306 (17)
C1	0.0683 (18)	0.0521 (18)	0.053 (2)	−0.0043 (14)	−0.0049 (15)	−0.0015 (15)
C2	0.080 (2)	0.061 (2)	0.078 (3)	0.0171 (16)	0.0202 (18)	0.0090 (18)
C3	0.077 (2)	0.064 (2)	0.066 (2)	0.0050 (16)	0.0190 (16)	0.0022 (17)
C4	0.0611 (17)	0.0522 (17)	0.0537 (19)	−0.0027 (14)	−0.0005 (14)	−0.0060 (15)
C5	0.105 (3)	0.067 (2)	0.056 (2)	0.0257 (18)	0.0040 (18)	−0.0072 (17)
C6	0.108 (3)	0.076 (2)	0.045 (2)	0.024 (2)	0.0099 (17)	−0.0013 (17)
C7	0.0653 (18)	0.0539 (18)	0.062 (2)	0.0012 (14)	0.0012 (15)	−0.0033 (16)
C8	0.0614 (17)	0.0513 (17)	0.0546 (19)	0.0060 (14)	0.0105 (14)	0.0014 (15)
C9	0.077 (2)	0.0543 (18)	0.060 (2)	−0.0126 (15)	0.0077 (16)	−0.0033 (16)
C10	0.0671 (18)	0.0615 (19)	0.0513 (19)	−0.0070 (15)	0.0037 (14)	−0.0017 (16)
C11	0.0494 (15)	0.0525 (17)	0.0538 (19)	0.0056 (13)	0.0128 (13)	−0.0009 (14)
C12	0.0622 (18)	0.0519 (18)	0.080 (2)	−0.0065 (14)	0.0004 (16)	0.0059 (17)
C13	0.0654 (18)	0.064 (2)	0.064 (2)	−0.0024 (15)	−0.0103 (15)	0.0039 (17)
C14	0.0526 (16)	0.0446 (16)	0.0484 (17)	−0.0006 (12)	0.0076 (12)	0.0003 (13)
C15	0.0623 (18)	0.0548 (17)	0.0516 (18)	−0.0093 (14)	0.0029 (14)	−0.0106 (14)
C16	0.0509 (17)	0.070 (2)	0.0550 (19)	−0.0057 (14)	−0.0043 (13)	−0.0053 (16)
C17	0.0552 (17)	0.0585 (18)	0.0493 (18)	0.0055 (14)	0.0007 (13)	0.0022 (14)
C18	0.0622 (18)	0.0495 (16)	0.0535 (18)	−0.0006 (14)	0.0003 (14)	−0.0087 (14)
C19	0.0496 (16)	0.0608 (18)	0.0568 (19)	−0.0082 (13)	0.0019 (13)	−0.0012 (15)
O4	0.1173 (19)	0.0697 (15)	0.0691 (16)	−0.0090 (14)	0.0174 (14)	−0.0028 (12)
C20	0.097 (3)	0.113 (3)	0.146 (4)	−0.026 (3)	0.022 (3)	0.017 (3)
C21	0.192 (5)	0.107 (3)	0.098 (3)	−0.055 (3)	−0.001 (3)	0.017 (3)

### Geometric parameters (Å, º)

**Table:**

S1—O2	1.443 (2)	C14—C15	1.390 (4)
S1—O3	1.435 (2)	C14—C19	1.382 (4)
S1—C11	1.758 (3)	C15—C16	1.363 (4)
S1—C14	1.737 (3)	C16—C17	1.389 (4)
O1—C1	1.355 (3)	C17—C18	1.398 (4)
O1—H1	0.8200	C18—C19	1.366 (4)
O4—C20	1.409 (5)	C2—H2	0.9300
O4—H4	0.8200	C3—H3	0.9300
N1—C7	1.260 (4)	C5—H5	0.9300
N1—C8	1.414 (4)	C6—H6	0.9300
N2—C17	1.363 (4)	C7—H7	0.9300
N2—H2B	0.8600	C9—H9	0.9300
N2—H2A	0.8600	C10—H10	0.9300
C1—C6	1.368 (4)	C12—H12	0.9300
C1—C2	1.378 (5)	C13—H13	0.9300
C2—C3	1.365 (5)	C15—H15	0.9300
C3—C4	1.382 (4)	C16—H16	0.9300
C4—C5	1.373 (4)	C18—H18	0.9300
C4—C7	1.464 (4)	C19—H19	0.9300
C5—C6	1.381 (4)	C20—C21	1.441 (7)
C8—C13	1.382 (4)	C20—H20A	0.9700
C8—C9	1.383 (4)	C20—H20B	0.9700
C9—C10	1.370 (4)	C21—H21A	0.9600
C10—C11	1.378 (4)	C21—H21B	0.9600
C11—C12	1.375 (4)	C21—H21C	0.9600
C12—C13	1.382 (4)		
			
O2—S1—O3	118.74 (13)	C17—C18—C19	120.1 (3)
O2—S1—C11	106.85 (12)	C14—C19—C18	120.7 (2)
O2—S1—C14	108.50 (14)	C1—C2—H2	120.00
O3—S1—C11	107.43 (12)	C3—C2—H2	120.00
O3—S1—C14	109.01 (12)	C4—C3—H3	119.00
C11—S1—C14	105.53 (13)	C2—C3—H3	119.00
C1—O1—H1	109.00	C4—C5—H5	119.00
C20—O4—H4	110.00	C6—C5—H5	119.00
C7—N1—C8	118.2 (2)	C5—C6—H6	120.00
C17—N2—H2B	120.00	C1—C6—H6	120.00
H2A—N2—H2B	120.00	N1—C7—H7	118.00
C17—N2—H2A	120.00	C4—C7—H7	118.00
C2—C1—C6	119.2 (3)	C8—C9—H9	120.00
O1—C1—C2	118.3 (3)	C10—C9—H9	120.00
O1—C1—C6	122.5 (3)	C11—C10—H10	120.00
C1—C2—C3	120.6 (3)	C9—C10—H10	120.00
C2—C3—C4	121.0 (3)	C13—C12—H12	120.00
C5—C4—C7	119.3 (3)	C11—C12—H12	120.00
C3—C4—C7	122.7 (3)	C8—C13—H13	120.00
C3—C4—C5	117.9 (3)	C12—C13—H13	120.00
C4—C5—C6	121.4 (3)	C14—C15—H15	120.00
C1—C6—C5	119.9 (3)	C16—C15—H15	120.00
N1—C7—C4	124.2 (3)	C17—C16—H16	119.00
C9—C8—C13	119.2 (3)	C15—C16—H16	119.00
N1—C8—C13	122.1 (2)	C17—C18—H18	120.00
N1—C8—C9	118.6 (3)	C19—C18—H18	120.00
C8—C9—C10	120.6 (3)	C18—C19—H19	120.00
C9—C10—C11	120.0 (3)	C14—C19—H19	120.00
C10—C11—C12	120.0 (3)	O4—C20—C21	114.9 (4)
S1—C11—C10	119.7 (2)	O4—C20—H20A	109.00
S1—C11—C12	120.3 (2)	O4—C20—H20B	109.00
C11—C12—C13	120.0 (3)	C21—C20—H20A	109.00
C8—C13—C12	120.1 (3)	C21—C20—H20B	109.00
S1—C14—C19	120.4 (2)	H20A—C20—H20B	108.00
C15—C14—C19	119.4 (2)	C20—C21—H21A	109.00
S1—C14—C15	120.0 (2)	C20—C21—H21B	109.00
C14—C15—C16	120.0 (3)	C20—C21—H21C	109.00
C15—C16—C17	121.0 (2)	H21A—C21—H21B	109.00
C16—C17—C18	118.7 (2)	H21A—C21—H21C	109.00
N2—C17—C18	120.8 (3)	H21B—C21—H21C	109.00
N2—C17—C16	120.5 (2)		
			
O2—S1—C11—C10	−33.3 (3)	C3—C4—C7—N1	18.7 (4)
O2—S1—C11—C12	146.1 (2)	C5—C4—C7—N1	−162.5 (3)
O3—S1—C11—C10	−161.7 (2)	C4—C5—C6—C1	2.3 (5)
O3—S1—C11—C12	17.7 (3)	N1—C8—C9—C10	−175.1 (3)
C14—S1—C11—C10	82.1 (2)	C13—C8—C9—C10	1.4 (4)
C14—S1—C11—C12	−98.5 (2)	N1—C8—C13—C12	174.5 (3)
O2—S1—C14—C15	22.7 (3)	C9—C8—C13—C12	−1.9 (4)
O2—S1—C14—C19	−162.3 (2)	C8—C9—C10—C11	1.0 (4)
O3—S1—C14—C15	153.4 (2)	C9—C10—C11—S1	176.5 (2)
O3—S1—C14—C19	−31.7 (3)	C9—C10—C11—C12	−2.9 (4)
C11—S1—C14—C15	−91.5 (2)	S1—C11—C12—C13	−176.9 (2)
C11—S1—C14—C19	83.5 (2)	C10—C11—C12—C13	2.5 (4)
C8—N1—C7—C4	−176.5 (2)	C11—C12—C13—C8	−0.1 (4)
C7—N1—C8—C9	−129.1 (3)	S1—C14—C15—C16	173.6 (2)
C7—N1—C8—C13	54.6 (4)	C19—C14—C15—C16	−1.4 (4)
O1—C1—C2—C3	−178.7 (3)	S1—C14—C19—C18	−173.9 (2)
C6—C1—C2—C3	0.3 (5)	C15—C14—C19—C18	1.1 (4)
O1—C1—C6—C5	177.3 (3)	C14—C15—C16—C17	−0.1 (4)
C2—C1—C6—C5	−1.7 (5)	C15—C16—C17—N2	−178.0 (3)
C1—C2—C3—C4	0.4 (5)	C15—C16—C17—C18	1.9 (4)
C2—C3—C4—C5	0.3 (5)	N2—C17—C18—C19	177.7 (3)
C2—C3—C4—C7	179.0 (3)	C16—C17—C18—C19	−2.2 (4)
C3—C4—C5—C6	−1.6 (5)	C17—C18—C19—C14	0.8 (4)
C7—C4—C5—C6	179.6 (3)		

### Hydrogen-bond geometry (Å, º)

**Table:**

*D*—H···*A*	*D*—H	H···*A*	*D*···*A*	*D*—H···*A*
O1—H1···O4^i^	0.82	1.89	2.698 (3)	171
N2—H2*A*···O3^ii^	0.86	2.30	3.105 (3)	156
N2—H2*B*···O2^iii^	0.86	2.21	3.026 (3)	157
O4—H4···N1^iv^	0.82	2.13	2.926 (3)	162

Symmetry codes: (i) −*x*+1, −*y*, −*z*+2; (ii) *x*−1, *y*, *z*; (iii) *x*−1/2, −*y*+1/2, *z*+1/2; (iv) −*x*+1, −*y*, −*z*+1.

## References

[bb1] Bocelli, G. & Cantoni, A. (1990). *Acta Cryst.* C**46**, 2257–2259.

[bb2] Bruker (2005). *SADABS* Bruker AXS Inc., Madison, Wisconsin, USA.

[bb3] Bruker (2009). *APEX2* and *SAINT* Bruker AXS Inc., Madison, Wisconsin, USA.

[bb4] Farrugia, L. J. (1997). *J. Appl. Cryst.* **30**, 565.

[bb5] Farrugia, L. J. (1999). *J. Appl. Cryst.* **32**, 837–838.

[bb6] Sheldrick, G. M. (2008). *Acta Cryst.* A**64**, 112–122. 10.1107/S0108767307043930 18156677

[bb7] Spek, A. L. (2009). *Acta Cryst.* D**65**, 148–155. 10.1107/S090744490804362X PMC2631630 19171970

